# Prevalence of Diabetes Mellitus and Its Risk Factors among Permanently Settled Tribal Individuals in Tribal and Urban Areas in Northern State of Sub-Himalayan Region of India

**DOI:** 10.1155/2014/380597

**Published:** 2014-05-06

**Authors:** Dhiraj Kapoor, Ashok Kumar Bhardwaj, Dinesh Kumar, Sunil Kumar Raina

**Affiliations:** ^1^Department of Medicine, Dr. Rajendra Prasad Government Medical College, Kangra, Himachal Pradesh 176001, India; ^2^Department of Community Medicine, Dr. Rajendra Prasad Government Medical College, Kangra, Himachal Pradesh 176001, India

## Abstract

*Background.* Effect of urban environment on the development of DM and its risk factors is studied with an ecological fallacy due to their study designs that formulate the background for the present study. *Objective.* To study the prevalence of DM and associated lifestyle related risk factors in traditional tribal individuals residing in tribal area and migrating persons of the same tribe to urban area of sub-Himalayan northern state of India. *Methodology.* Population based cross-sectional study. *Results.* A total of 8000 individuals (tribal: 4000; urban: 4000) were recruited. Overall, among urban tribes the prevalence of central obesity (59.0%), overweight (29.3%), stage 1 (22.8%) and stage 2 (5.3%) hypertension, and DM (fasting: 7.8%; OGTT: 8.5%) (*P* = 0.00) was significantly higher than the tribes of tribal area. Based on OGTT, the prevalence of DM was found to be 9.2% among central obese tribes of urban area and 6.7% of tribal area (*P* = 0.00). DM showed a significant high prevalence among urban tribes with prehypertension (urban: 8.3%; tribal: 2.9%; *P* = 0.00), and stage 1 (urban: 14.1%; tribal: 8.7%; *P* = 0.00) and stage 2 (urban: 17.5%; tribal: 13.9%; *P* = 0.59) hypertension. *Conclusion*. Urban environment showed a changing lifestyle and high prevalence of DM among tribal migrating urban tribes as compared to traditional tribes.

## 1. Introduction

Emerging trend of diabetes mellitus (DM) is observed worldwide, as by 2025, its prevalence is projected to be 6.3%, which is a 24.0% increase compared with 2003. There will be 333 million (a 72.0% increase) diabetics by 2030 in individuals of 20 to 79 years of age. The developing world (mainly central Asia and Sub-Saharan Africa) accounted for 141 million people with diabetes (72.5% of the world total) in 2003 [1]. Environmental factors like obesity (central or general), physical inactivity, and diet (saturated fats and transfatty acids) and socioeconomic factors are responsible for development of DM [2–6]. Diet rich in polyunsaturated fats and long chain omega-3 fatty acids reduces the risk for DM [7].

Along with the rising trend of DM, rapid urbanization has been observed as from 2008 to 2030 the global urban population will increase by 1.6 billion people (from 3.3 billion to 4.9 billion). While during the same period the rural population is going to reduce by 28 million. This demographic transition will largely take place in developing countries (particularly in Asia and Africa), as by 2030, the developing world population will constitute more than 80% of the world's urban population [2, 3]. United Nations (UN) recognized that urbanization has health implications in terms of changing epidemiology of communicable and noncommunicable diseases (NCDs) including respiratory conditions, heart disease, DM, cancers, and many more [8, 9].

Urbanization and technology use led to Westernization due to cultural acculturation. This has been associated with the high prevalence of DM in many indigenous populations and in developing economies [10], whereas surrogate markers for improved socioeconomic status, such as level of attained education and income, found to be inversely associated with diabetes in high-income countries [11]. Throughout the process of development and urbanization, national economies are shifting away from physically active economic activities like farming, mining, forestry, and so forth to a more sedentary, often office-based, occupations [12]. So far, urbanization was quantified and its effect on the development of chronic diseases and its risk factors were studied at population level [13–15]. Such analysis at population level has inherent ecological fallacy and in order to investigate the effect of urban environment on individual the present study was planned. For its purpose a traditional tribe who were residing in tribal area and people of same tribe who were sharing the same culture and lifestyle but migrated and permanently settled in urban area were selected to study the prevalence of DM and associated lifestyle related risk factors.

## 2. Methodology

The population based cross-sectional study was carried out for three years (2008–2010) in tribal (Gaddi) community residing in tribal and urban areas of Himachal Pradesh, India. This community was notified as a tribal community by the government of Himachal Pradesh. This community was chosen to study as they represent traditional hilly people who settled in mountainous terrain centuries ago rendering a seminomadic life and were migrating between alpine Himalayas and Himalayan foothills. They usually stay in difficult terrain and face harsh weather conditions with limited access to modern facilities. For the study purpose, the tribal area of the community was Bharmour of Chamba district and urban area was Dharamshala of Kangra district of Himachal Pradesh. Bharmour was an area (tribal) where people of Gaddi population initially settled in mountains. It still has a very high population for Gaddi people (total population as per 2001 census was 22723 and 85% are of Gaddi (tribal) community). The Gaddi population of Dharamshala was included as they share same the ancestry as the Gaddi's of Bharmour but settled in urban area and changed their lifestyle and their means of livelihood.

A total of 4000 tribes from 30 random clusters of tribal (Bharmour) and urban areas (Dharamshala) were selected, respectively. Revenue village in tribal and Municipal Corporation ward in urban area was unit of cluster. The sample distribution in each of these areas was based on the proportion of the population in that particular area. A probabilistic proportionate sampling method was used to select the number of individuals in each area. Further, within each area, every third lane/road, following the right hand rule, was surveyed first and whole village/ward was covered by conducting a house to house survey. Such approach was chosen as it enabled equitable distribution of entire study population with less sampling error. Another advantage was the simplicity of involved administrative procedures. The eligible subjects were both men and women of ≥20 years of age, irrespective of their socioeconomic status or occupation. The final sample contained a total of 8,000 subjects. Ethical committee approval had been obtained for the study and informed consent was obtained from all the study subjects.

Study subjects were assessed using a case recording format including information for socioeconomic status, physical activity (duration of work of >90, 60–90, 30–59, and <30 minutes per day as heavy, moderate, mild, and sedentary, resp.), diet, smoking, and alcohol use. After information recording an anthropometric assessment (height, weight, and waist and hip circumference) was done. Blood pressure was taken after a 10-minute rest with standard cuffs for adults fitted with a mercury sphygmomanometer in sitting position. Blood pressure was taken twice (in a gap of 10 minutes) and average value was considered.

A 12-hour fasting blood sample (10 mL, in two aliquots—one aliquot in EDTA and another without anticoagulant) was collected from each individual by venipuncture maintaining aseptic conditions. In an individual DM was assessed by using fasting blood glucose and oral glucose tolerance test (OGTT). OGTT was performed using 75 g glucose in the field settings and diagnostic criteria laid by Indian Council of Medical Research (ICMR) were used to diagnose DM (Table 1).

## 3. Results

A total of 4000 individuals were recruited in each of the tribal and urban areas and recruitment was high (51.8%) for males of tribal area (*P* = 0.00). Among recruited individuals of tribal tribes, it was found that significantly more individuals (45.3%) were less than 35 years of age, whereas significantly large proportion of urban tribe was of 36 to 50 (34.0%) and more than 65 (7.4%) years of age. The marriage rate was 79.4% and 86.2% among tribes of tribal and urban areas, respectively (*P* = 0.00). The literacy rate was observed to be significantly more (66.0%) among tribes of urban area (*P* = 0.00). Significantly, most of tribal tribes were students (11.8%), farmers (24.1%), and retired personnel from a government job (8.8%), and among urban tribes most were engaged in the private (33.3%) and government (5.3%) sectors (*P* = 0.00). A large fraction of individuals (mostly women) were engaged in domestic (daily household) activities, but more significantly (50.0%) in urban area. Almost all tribes were of nonvegetarian dietary nature in both tribal (94.1%) and urban (93.6%) areas (*P* = 0.35). When assessed for substance abuse, tobacco smoking (35.1%) was significantly more among tribal tribes (*P* = 0.00). Alcohol was indifferently prevalent (*P* = 0.15) among tribal (33.0%) and urban (34.5%) tribes. When assessed for level of physical activity in daily routine, it was found that significantly most of the tribes of the tribal area were engaged in moderate (62.8%) to heavy (23.0%) physical activity, and urban tribes were in sedentary (9.0%) to mild (8.6%) type of activity (Table 2).

It was found that significantly most of the urban tribes had high average level of age (42.4 years), BMI (21.4 Kg/m^2^), WHR (0.88), blood pressure (134/71), blood sugar (OGTT; 171.9 g/dL), and consumption of alcohol (293.2 mL/day). In both males and females of urban area, significantly high mean levels were observed for age, BMI, WHR, blood pressure, blood sugar (both fasting and OGTT in females and OGT in males), and alcohol consumption (Table 3). In urban tribes of up to 65 years of age, the mean levels were significantly high for BMI, WHR, blood glucose (fasting and OGTT), and alcohol (*P* = 0.00) consumption. In addition to these variables, urban tribes of 36 to 65 years of age also had significantly high (*P* = 0.00) mean levels of blood pressure (systolic and diastolic). Urban tribes of more than 65 years of age had a significantly high level of mean BMI, blood pressure, and alcohol consumption, whereas tribal tribes of the same age group had high levels of fasting blood glucose (*P* = 0.00). Based on OGTT, the mean level of glucose was observed to be indifferent among individuals of more than 65 years of age in both areas (tribal: 179.2; urban: 177.2 mg/dL) (Table 4).

Overall, in urban tribe the prevalence of central obesity (59.0%), overweight (29.3%), stage 1 (22.8%) and stage 2 (5.3%) hypertension, and DM (fasting: 7.8%; OGTT: 8.5%) (*P* = 0.00) was significantly higher than in tribes of tribal area. The prevalence of the above-mentioned variables was found to be significantly more among both males and females in tribes of urban area (Table 5). It was also observed significantly more among tribal individuals up to 65 years of age of urban area. Individuals of more than 65 years of age had significantly high prevalence for overweight, obesity, and stage 2 hypertension only. Stage 1 hypertension was significantly and DM (based on FBG and OGTT) was insignificantly more in tribal area (Table 6). The prevalence of prehypertension, IFG, and IGT was found to be more among tribes of the tribal area for both males and females and across all the age groups (Tables 5 and 6). In the area, a rising prevalence of hypertension (stage 1 and stage 2) and DM (FBG and OGTT) was observed in individuals from age group of <35, 36 to 50, and 51 to 65 years (Table 6).

The prevalence of central obesity was observed up to 60.0% in all age groups of urban tribe. Among urban tribes, an overweight prevalence was observed to be the highest (31.9%) among individuals of 36 to 50, 31.2% among 51 to 65, 27.5% among less than 35, and 21.5% among more than 65 years of age. Prevalence of hypertension (combined stage 1 and 2) was high among urban tribe of more than 65 (54.0%), followed by 51 to 65 (48.3%), and 36 to 50 years of age (34.2%). The prevalence of DM based on fasting (12.0%) and OGTT (12.4%) was observed to be insignificantly high among tribal individuals of the tribal area among more than 65 years of age, whereas the prevalence was insignificantly high in tribes of urban area of 51 to 65 years of age for DM (FBG: 12.3%; OGTT: 12.7%) (Table 6).

Prevalence of DM was also analyzed among individuals with obesity, hypertension, physical activity, and substance abuse. Based on OGTT, the prevalence of DM was found to be 9.2% among central obese tribes of urban area and 6.7% of tribal area (*P* = 0.00). Among overweight tribes of urban area DM was 11.3% and 2.1% in tribal area (*P* = 0.00). DM showed a significant high prevalence among urban tribes with prehypertension (urban: 8.3%; tribal: 2.9%; *P* = 0.00) and stage 1 (urban: 14.1%; tribal: 8.7%; *P* = 0.00) and stage 2 (urban: 17.5%; tribal: 13.9%; *P* = 0.59) hypertension. Similarly, a rising prevalence of DM was observed among tribes carrying out heavy to sedentary levels of physical activity of both areas, but it was significantly more (*P* = 0.00) in urban area. DM was highly prevalent among tribal tribes (overall: 30.2%) with sedentary (urban: 32.6%; tribal: 26.2%) lifestyle of both areas (*P* = 0.10). Although more than 90.0% of tribal tribes of both areas had nonvegetarian type of diet, DM was 8.7% in urban area and 4.0% in tribal area (*P* = 0.00). As diet, smoking, and alcohol were equally prevalent among tribal tribes of both areas, tribal tribes of urban area had 11.5% prevalence of DM among smokers and 11.9% among alcohol users (*P* = 0.00) (Table 7).

## 4. Discussion

Urbanization as a process is simultaneously changing the daily lifestyle in the form of increase in fat consumption, physical inactivity, and substance abuse with associated risk of development of chronic diseases like hypertension and DM. It is a cause of concern in developing countries as it shares a significant proportion of the world population and so the morbidity and mortality due to chronic diseases [16, 17]. Effect of an urban environment onto the lifestyle pattern was studied and showed that the individual who resided in urban environment had two times more chance to become overweight and obese [18]. Evidence from South Africa observed that the hypertension was positively associated with urbanization [19]. In India, it was found that the prevalence of DM was two and half times higher in urban than in rural area [20].

Globally, age-standardized prevalence of DM was found to be 9.8% in men and 9.2% in women with observed regional disparity, as a high prevalence of DM was found in South Asia, Latin America, the Caribbean, Central Asia, North Africa, and the Middle East [21]. Disparity within country was observed in India as in urban areas the prevalence of DM is from 5.9% to 12.1% (North: 8.6% to 11.6%; South: 13.5% to 19.5%) [22, 23]. A nationwide survey across India showed 1.3% prevalence of self-reported DM, which was more in men (1.5%) as compared to women (1.0%) [23]. In addition to urban India, rural India also found high prevalence of DM (about 2.0% to 10.0%) [22]. A systematic review for DM in tribal population of India observed a ranging prevalence of 0.7% to 10.0%, with a final estimate of 5.9% [23]. Urbanization at the cost of shrinking of rural areas led to onset of lifestyle related chronic diseases. As there was no evidence for effect of urban environment onto tribal population, the present study was done to assess the effect of urban environment on tribal population for development of lifestyle related risk factors and DM. In present study, the prevalence of DM was found to be significantly high among tribal tribes of urban area (8.5%) than of tribal (4.5%) area, which is less than of 5.9% observed in a systematic review [23]. Among tribal tribes of urban area, the prevalence of DM was 7.8% and 9.1% among males and females, respectively (*P* = 0.00).

As evidence from urban India showed, an observed prevalence of DM and IGT was 12.1% and 14.0%, respectively [24]. Systematic review from tribal population observed ranging prevalence of IGT from 6.6% to 12.9% [23]. Current study observed a very high prevalence of IGT (around 90.0%) in both the urban and tribal areas but significantly more in tribal tribes and among tribal males of tribal area (Table 5). Present study observed high prevalence of IGT across all the age groups and is in contrary to the studies from urban India which observed a high prevalence of IGT among individuals of less than 40 years of age [24, 25]. Evidence showed that DM was 3.7% in urban and 2.1% in rural population of 26 to 32 years of age [26]. In current evidence, the prevalence of DM was observed to be 11.0% in urban area and 3.3% in tribal area of 36 to 50 years of age and, among all cases of DM (based on OGTT), 32.3% of tribal and 63.0% of urban areas had ages less than 50 years (data not shown). The early onset of DM was also suggested by a national survey in India which found that half of diabetics developed disease in less than 50 years of age [24]. The observed higher prevalence of IGT in present study than from systematic review possibly may be due to different populations (selection bias) like residing at high altitude with high consumption of nonvegetarian diet.

It had been evident that the Asian Indians are more susceptible to risk factors like age, adiposity (based on BMI), and central obesity (WHR) [27, 28]. Despite the low BMI among Asian Indians as compared to other ethnic groups, BMI was strongly associated with glucose tolerance [24]. The risk of DM was observed to be significant for urban population with BMI more than 23 kg/m^2^ [29, 30]. It had also been evident from studies across different parts of India, migrant Indians, and other Asian individuals [30–32]. Present study showed that the DM prevalence was high among overweight (tribal: 2.1%; urban: 11.3%) and obese (tribal: 16.7%; urban: 23.1%) tribal tribes of both areas (Table 6). It was also evident that risk of age adjusted DM starts to increase with the BMI in both males and females. Also in both areas present study showed that there was an increasing trend of DM prevalence among tribal tribes below 65 years of age (Table 6). Abdominal adiposity especially visceral fat was observed to increase the insulin resistance and so the risk for DM and hyperlipidemia [33]. Present study found that the prevalence of DM was 4.0% in tribal and 9.2% in urban tribes with central obesity. Tribes of tribal area showed significantly low level of mean WHR and lower BMI as compared to urban area (Table 3). As for the tribal area of present study, rural populations had also been observed with a significant lower BMI than an urban population with similar WHR [34]. It can be due to engagement of tribes of tribal area into moderate to heavy type of physical activity (grazing animals, harvesting, plowing, etc.) more than their urban counterpart (Table 2), and DM was less prevalent in tribal area, those who were engaged in moderate to heavy physical activity (Table 6). Current study also showed that, among underweight (BMI < 18.5 kg/m^2^) tribes, the prevalence of DM was 10.2% and 4.5% in tribal and rural urban areas, respectively (Table 7).

Apart from DM, hypertension is a common chronic morbidity which affects population with the common lifestyle and diet related risk factors. It is also known that hypertension is common morbidity among patients with DM. A retrospective chart analysis of 2227 individuals of type 2 diabetes in a hospital showed that the prevalence of hypertension was 60.2%, 76.5%, and 85.8% at blood pressure thresholds of 140/90, 130/85, and 130/80 mm Hg, respectively [35]. Another study showed an association of DM among diagnosed cases of hypertension [36]. A cross-sectional study in 10 states in India observed coexistence of DM and hypertension in 20.6% of patients; the prevalence of prehypertension was observed to be 60.0% [37]. Among studied tribal tribes in present study the overall prevalence of DM was 10.4% in stage 1 and 16.9% in stage 2 hypertension (Table 7). It was observed to be more among tribes of urban (stage 1: 14.1%; stage 2: 17.5%) as compared to tribal (stage 1: 8.7%; stage 2: 13.9%) areas. In India, the overall prevalence of hypertension was about 40.0%, more in urban (63.2%) than in rural (36.8%) areas. Among adults the prevalence of hypertension varied from 20.0% to 40.0% in urban India and 12.0% to 17.0% in rural India [36]. Current study observed that prevalence of hypertension (>120/80 mm of Hg) was 91.5% (tribal: 93.3%; urban: 90.6%) among studied tribes.

Smoking along with DM causes an extra risk for macro- and microvascular complications which further increases cardiovascular morbidity and mortality. It was also stated that smoking increases the risk for DM itself. In a follow-up study of Asian cohort, smokers were found to have an equivalent risk of all-cause mortality as to an elevation of blood glucose by an average of 41 mg/dL for the cohort in general and 68 mg/dL for the diabetes in particular [38]. Smoking was found to cause substantial changes in insulin sensitivity among patients with noninsulin DM. A meta-analysis showed that the prevalence of smoking was 33.3% among diabetic patients as compared to 27.0% among normal subjects [39]. An assessment showed that both current and past smoking are associated with a risk of diabetes mellitus essentially in men, but much less in women with the relationship between fasting glucose and smoking [40]. In present study, the overall prevalence of DM was 7.1% among smokers and was significantly high among tribal tribes of urban areas (11.5%) than of tribal (3.8%) areas.

Effect of alcohol consumption onto the development of DM showed ambiguous conclusions. A prospective population based study observed association of alcohol consumption and risk of DM [41]. In another study, the risk of DM was observed to be more among patients with liver cirrhosis due to alcohol consumption [42]. However, population based survey in three areas showed no effect of independent alcohol consumption on the development of DM [43]. Japanese evidence showed that the alcohol was a risk factor for DM among individuals with low BMI [44]. A review of prospective studies concluded that there is a delicate balance between harmful and beneficial effects of alcohol onto the incidence of diabetes. In moderate amounts, drinking alcohol is associated with a reduced risk of diabetes, whereas in higher amounts with an increased risk [45]. A meta-analysis suggested that there was an approximately 30% reduction in the risk of type 2 diabetes in moderate alcohol consumers, whereas no risk reduction is observed in heavy consumers (≥48 g/day) [46]. In present study, the prevalence of DM was 7.6% (tribal: 3.2%; urban: 11.9%) among tribal tribes consuming alcohol which was significantly high in urban area (Table 7).

Rapid urbanization in many regions of the world like Sub-Saharan region had observed an association with the emergence of noncommunicable diseases (NCDs). Evidences demonstrated that there was a positive association of rural-urban gradient to the emergence of environmental risk factors [9]. A study carried in Sri Lanka observed a clear relationship between urbanicity and common modifiable risk factors for chronic diseases [13]. Obesity, dietary changes (particularly increase in dietary fat intake), and physical inactivity are widely accepted as lifestyle risk factors for NCDs which increases as environments become more urban [16]. Examples from the developing world, like Mexico, South Africa, Malaysia, Thailand, India, and Tanzania, also showed that there was an association between urban living and the risk of development of diabetes mellitus, overweight and obesity, and hypertension [47–52]. A study done in Cameroon found that there was an effect of time spent living in a developed (urban) environment area on a number of chronic disease risk factors [18]. A study of adults in the North Western province of South Africa found that blood pressure was correlated positively with level of urbanization [19]. A large risk factor surveillance study conducted in India found that the prevalence of diabetes was two and a half times higher in urban areas when compared to rural areas [20]. The “nutrition transition” has been observed as a recent and rapid change in the diet among populations of many countries with increases in the consumption of foods sourced from animals, caloric sweeteners, and fat along with consumption of fast foods [53–56].

Evidence showed a positive and independent association with age, BMI, WHR, family history of diabetes, monthly income, and sedentary physical activity [24]. In current study the prevalence of DM was observed to be high among tribal tribes with lifestyle related risk factors like obesity, hypertension, nonvegetarian diet, and physical inactivity. Prevalence of DM among tribal tribes positive for these risk factors was observed to be significantly high among tribal tribes of urban area than of tribal area. It can be suggested that tribes changed their behavior in an urban environment with the development of lifestyle related risk factors and so the high prevalence of DM. There is a need to formulate and implement the cultural specific lifestyle and nutritional interventions to reduce the type 2 DM.

## Figures and Tables

**Table 1:** Criteria of Indian Council of Medical Research (ICMR) for diagnosis of diabetes and glucose tolerance.

Normoglycemia	IFG/IGT	Diabetes
FPG < 110 mg/dL	FPG ≥ 110 and <126 mg/dL (IFG)	FPG ≥ 126 mg/dL

2-h PG < 140 mg/dL	2-h PG ≥ 140 and <200 mg/dL (IGT)	2-hour PG ≥ 200 mg/dL symptoms of diabetes and causal plasma glucose concentration ≥200 mg/dL

IFG: impaired fasting glucose; IGT: impaired glucose tolerance test; FPG: fasting plasma glucose; 2-h PG: 2-hour postload glucose test (oral glucose tolerance test).

**Table 2:** Distribution of baseline characteristics among tribes of urban and tribal areas, Himachal Pradesh, 2011-2012.

Characteristics	Tribal tribes (4000)	Urban tribes (4000)	*P* value	Both (8000)
Male	51.8	44.7	0.00	48.2
Literate	60.2	66.0	0.00	63.1
Married	79.4	86.2	0.00	82.8
Age group				
<35	45.3	39.6	0.00	42.4
36–50	31.2	34.0	0.00	32.6
51–65	17.3	18.9	0.06	18.1
>65	6.2	7.4	0.03	6.8
Occupation				
Student	11.8	5.7	0.00	8.7
Farmer	24.1	4.0	0.00	14.0
Private	11.1	33.3	0.00	22.2
Domestic	42.8	50.0	0.00	46.4
Government	1.5	5.3	0.00	3.4
Retired	8.8	1.8	0.00	5.3
Nonvegetarian	94.1	93.6	0.35	93.9
Smoking	35.1	25.4	0.00	30.3
Alcohol	33.0	34.5	0.15	33.8
Physical activity				
Heavy	23.0	7.3	0.00	15.2
Moderate	62.8	59.8	0.00	61.3
Mild	8.6	19.3	0.00	14.0
Sedentary	5.3	9.0	0.00	7.2

**Table 3:** Gender distribution of mean levels of anthropometry, blood glucose, and alcohol consumption among tribes of urban and tribal areas, Himachal Pradesh, 2011-2012.

Characteristics (mean)	Male			Female			Total		
	Tribal tribes (2070)	Urban tribes (1788)	*P* value	Tribal tribes (1930)	Urban tribes (2212)	*P* value	Tribal tribes (4000)	Urban tribes (4000)	*P* value
Age (years)	39.8	42.5	0.00	40.5	42.3	0.69	40.2	42.4	0.00
BMI (kg/m^2^)	21.0	21.8	0.00	20.5	20.9	0.00	20.8	21.4	0.00
WHR	0.87	0.89	0.00	0.87	0.88	0.00	0.87	0.88	0.00
BP (mm of Hg)	130.0	135/24	0.00	130/18	134/28	0.00	130/41	134/71	0.00
FBG (mg/dL)	88.2	88.5	0.65	89.7	90.2	0.00	88.9	89.4	0.28
OGTT (mg/dL)	157.6	171.3	0.00	159.7	172.4	0.00	158.6	171.9	0.00
Alcohol (mL)	250.3	293.5	0.00	217.3	273.7	0.02	249.7	293.2	0.00

**Table 4:** Age group distribution of mean levels of anthropometry, blood glucose, and alcohol consumption among tribes of urban and tribal areas, Himachal Pradesh, 2011-2012.

Characteristics (mean)	Age group (years)											
	<35			36–50			51–65			>65		
	Tribal tribes (1811)	Urban tribes (1583)	*P* value	Tribal tribes (1247)	Urban tribes (1362)	*P* value	Tribal tribes (693)	Urban tribes (757)	*P* value	Tribal tribes (249)	Urban tribes (298)	*P* value
BMI (kg/m^2^)	20.7	21.3	0.00	21.0	21.5	0.00	20.8	21.4	0.00	19.7	20.7	0.00
WHR	0.87	0.88	0.00	0.87	0.88	0.00	0.87	0.88	0.00	0.88	0.88	0.44
BP (mm of Hg)	128/27	130/32	0.17	130/18	134/76	0.00	133/84	140/89	0.00	103/86	92/91	0.00
FBG (mg/dL)	83.4	85.0	0.00	88.4	90.8	0.00	94.8	98.9	0.00	103.8	92.9	0.00
OGTT (mg/dL)	151.8	164.9	0.00	156.9	174.8	0.00	171.8	179.2	0.00	179.2	177.2	0.62
Alcohol (mL)	258.6	284.4	0.00	256.5	293.4	0.00	237.3	301.5	0.00	198.1	291.3	0.00

**Table 5:** Gender distribution for anthropometry, blood glucose, and alcohol consumption among tribes of urban and tribal areas, Himachal Pradesh, 2011-2012.

Characteristics (%)	Male			Female			Total		
	Tribal tribes (2070)	Urban tribes (1788)	*P* value	Tribal tribes (1930)	Urban tribes (2212)	*P* value	Tribal tribes (4000)	Urban tribes (4000)	*P* value
Central obesity	30.2	46.9	0.00	57.4	68.8	0.00	43.3	59.0	0.00
BMI (kg/m^2^)									
<18.5	5.3	4.9	0.58	10.0	10.1	0.93	7.6	7.8	0.73
23.0–27.5	16.9	43.3	0.00	8.8	18.0	0.00	13.0	29.3	0.00
>27.5	0.2	0.3	0.82∗	0.4	0.4	0.96	0.3	0.3	1.00
HTN (mm of Hg)									
Prehypertension	81.5	59.8	0.00	75.9	56.8	0.00	78.8	58.2	0.00
Stage 1	8.8	23.8	0.00	11.8	22.0	0.00	10.2	22.8	0.00
Stage 2	0.7	5.1	0.00	1.1	5.5	0.00	0.9	5.3	0.00
FBG (mg/dL)									
IFG (110–126)	1.9	0.3	0.00	3.1	1.0	0.00	2.4	0.7	0.00
DM (>126)	3.3	7.0	0.00	4.6	8.4	0.00	3.9	7.8	0.00
OGTT (mg/dL)									
IGT (140–200)	94.5	84.9	0.00	92.4	81.6	0.00	93.5	83.1	0.00
DM (>200)	3.5	7.8	0.00	4.8	9.1	0.00	4.1	8.5	0.00

^*^Yates Corrected Chi-square test.

**Table 6:** Age group distribution of mean levels of anthropometry, blood glucose, and alcohol consumption among tribes of urban and tribal areas, Himachal Pradesh, 2011-2012.

Characteristics	Age group (years)											
	<35			36–50			51–65			>65		
	Tribal tribes (1811)	Urban tribes (1583)	*P* value	Tribal tribes (1247)	Urban tribes (1362)	*P* value	Tribal tribes (693)	Urban tribes (757)	*P* value	Tribal tribes (249)	Urban tribes (298)	*P* value
Central obesity	42.7	59.3	0.00	39.1	51.5	0.00	48.5	58.1	0.00	54.2	57.4	0.45
BMI (kg/m^2^)												
<18.5	6.4	6.8	0.62	4.3	7.0	0.00	9.3	8.6	0.66	27.7	14.8	0.00
23.0–27.5	13.2	27.7	0.00	13.8	31.9	0.00	12.6	31.2	0.00	8.8	21.5	0.00
>27.5	0.1	0.2	0.88∗	0.4	0.5	0.67	0.7	0.4	0.63∗	0.0	0.7	0.00
HTN (mm of Hg)												
Prehypertension	84.8	72.2	0.00	83.4	54.9	0.00	67.5	42.4	0.00	44.2	38.6	0.18
Stage 1	2.0	7.5	0.00	8.4	31.9	0.00	23.5	35.9	0.00	42.2	29.5	0.00
Stage 2	0.3	0.8	0.03	0.9	2.3	0.00	1.6	12.4	0.00	3.6	24.5	0.00
FBG (mg/dL)												
IFG (110–126)	0.6	0.5	0.69	0.8	0.7	0.84	5.8	1.1	0.00	14.9	0.7	0.00
DM (>126)	0.6	3.2	0.00	3.1	10.3	0.00	11.1	12.3	0.48	12.0	9.4	0.31
OGTT (mg/dL)												
IGT (140–200)	96.9	87.8	0.00	94.2	80.5	0.00	86.7	78.2	0.00	83.9	81.9	0.52
DM (>200)	0.7	4.1	0.00	3.3	11.0	0.00	11.5	12.7	0.50	12.4	10.1	0.37

^*^Yates Corrected Chi-square test.

**Table 7:** Prevalence of DM among tribes exposed to overweight, hypertension, physical activity, diet, and substance abuse in tribal and urban areas of Himachal Pradesh, 2011-2012.

Characteristics	Tribal	Urban	DM (FG)				DM (OGTT)			
			Tribal tribes	Urban tribes	*P* value	Both	Tribal tribes	Urban tribes	*P* value	Both
Central obesity	1733	2361	4.1	8.6	0.00	6.7	4.0	9.2	0.00	7.0
BMI (Asian)										
<18.5	322	311	9.3	4.2	0.01	6.8	10.2	4.5	0.00	7.4
18.5–22.9	3148	2504	3.5	6.6	0.00	4.9	3.7	7.6	0.00	5.5
23.0–27.5	518	1172	2.7	11.0	0.00	8.5	2.1	11.3	0.00	8.5
>27.5	12	13	16.7	23.1	0.92∗	20.0	16.7	23.1	0.92∗	20.0
Hypertension										
Normal	401	548	2.7	3.8	0.35	3.4	2.7	5.8	0.02	4.5
Prehypertension	3153	2327	2.8	7.6	0.00	4.8	2.9	8.3	0.00	5.2
Stage 1	410	913	13.2	8.3	0.00	9.8	8.7	14.1	0.00	10.4
Stage 2	36	212	13.9	17.0	0.64	16.5	13.9	17.5	0.59	16.9
Physical activity										
Heavy	922	293	1.8	6.1	0.00	2.9	2.1	6.5	0.00	3.1
Moderate	2512	2392	2.9	4.8	0.00	3.9	3.0	5.7	0.00	4.3
Mild	344	774	3.8	8.4	0.00	7.0	3.8	8.7	0.00	7.2
Sedentary	214	362	25.2	30.4	0.18	28.5	26.2	32.6	0.10	30.2
Nonvegetarian	3764	3745	3.9	8.0	0.00	5.9	4.0	8.7	0.00	6.4
Smoking	1405	1017	3.8	10.9	0.00	6.8	3.8	11.5	0.00	7.1
Alcohol	1321	1383	3.3	7.2	0.00	5.3	3.2	11.9	0.00	7.6

^*^Yates Corrected Chi-square test.
